# Gut microbiome shift in long COVID: impact of disease and montelukast treatment

**DOI:** 10.7189/jogh.16.04164

**Published:** 2026-05-15

**Authors:** Paula Camps-Massa, Judit Pérez-Mormeneu, Daiana Guevara-Nuñez, Lucía Saiz-Escobedo, Laura Calatayud, Aida González-Díaz, Albert Sanllorente, Vanesa Vicens-Zygmunt, Salud Santos, Rosa Morros, Betlem Salvador-González, Mª Ángeles Domínguez, Sara Martí, E-SPERANZA study group, Jesús Almeda-Ortega, Sara Contreras-Martos, Sara Bonet Monne, Gemma Alvarez Muñoz, Francisco Mera-Cordero, Nancy Eydis Castillo Elinan, Beatriz Navarro Martín, Maria Jesús Gallardo Guerra, Jenifer Botanes Iglesias, Sofía Centeno Manotas, Maria Antonia Coll Bosch, Cristina Armengol Mercade, Elena Barquero Bardon, Marta Via Vidal, Encarnación Magraner Esteve, Maria Teresa Ortiz Lupiañez, Arnau Segura Anducas, Francisco Javier Calero Ribera, Cristina López Ramírez, Alex Trepat González, Josefa Pérez Ruiz, Isabel Zamora Casas, Carles Rubio Ripollès, Ramon Monfà Escolà, Ana García Sangenís, Oriol Cunillera Puértolas, Cristina Miranda Jiménez, Anna Cortes Bosch de Basea, Anna Ferrer Artola

**Affiliations:** 1 Microbiology Department, Hospital Universitari de Bellvitge – Bellvitge Biomedical Research Institute (IDIBELL) – University of Barcelona (UB), Barcelona, Spain; 2 Department of Pathology and Experimental Therapeutics, University of Barcelona (UB), Barcelona, Spain; 3 Research Network for Respiratory Diseases (CIBERES), Instituto de Salud Carlos III, Madrid, Spain; 4 Pneumology Department, Hospital Universitari de Bellvitge – Bellvitge Biomedical Research Institute (IDIBELL) – University of Barcelona (UB), Barcelona, Spain; 5 Primary Care Research Unit Metropolitana Sud, Institut Català de la Salut, El Prat de Llobregat, Spain; 6 Institut Universitari de Recerca en Atenció Primària Jordi Gol i Gurina, Barcelona, Spain; 7 Department of Pharmacology and Therapeutics, Universitat Autònoma de Barcelona (UAB), Bellaterra, Spain; 8 Research Network for Infectious Diseases (CIBERINFEC), Instituto de Salud Carlos III, Madrid, Spain; 9 Department of Medicine, University of Barcelona (UB), Barcelona, Spain

## Abstract

**Background:**

Long COVID-19 is a post-infectious syndrome with persistent symptoms that can involve multiple organ systems. Evidence suggests that SARS-CoV-2 infection may disrupt gut microbiome composition, potentially contributing to long-term effects. As treatment remains symptom-based, interest has grown in repurposing drugs like montelukast. However, non-antibiotic medications may also alter gut microbial communities, raising questions about their impact. Here, we compare gut microbiota between long COVID patients and healthy controls and examine how montelukast treatment affects microbial composition.

**Methods:**

We analysed stool samples from long COVID patients and healthy controls using 16S rRNA gene sequencing (Illumina MiSeq). We evaluate alpha (Shannon) and beta (Bray–Curtis) diversity, followed by relative abundance and linear discriminant effect size analysis, to identify differentially abundant taxa. This proof-of-concept study included a cross-sectional comparison and a longitudinal analysis of montelukast-treated patients *vs*. placebo.

**Results:**

Cross-sectional analysis revealed a significant structural reorganisation of the gut microbial community in long COVID patients, although overall species richness was largely maintained. Linear discriminant effect size analysis revealed that this architectural shift was driven by an enrichment of Firmicutes (*Agathobacter* and *Faecalibacterium* genera) in the long COVID group, while healthy controls were characterised by higher abundances of the phyla Verrucomicrobiota and Actinobacteriota, as well as genera *Alistipes* and *Akkermansia*. Longitudinal analysis demonstrated that the broader community structure remained stable in both groups; however, montelukast treatment led to a specific enrichment of the genus *Dialister*, suggesting targeted and potentially transient effects without disrupting the overall microbial landscape.

**Conclusions:**

Long COVID is characterised by a significant restructure of the gut ecosystem. This qualitative dysbiosis reflects a shift in homeostatic balance, where the core microbial community remains present, but its proportions are altered. Short-term montelukast treatment shows a minimal impact on the microbial landscape, suggesting treatment does not further destabilise the gut environment. These findings highlight the specific and targeted nature of gastrointestinal involvement in long COVID.

COVID-19, caused by severe acute respiratory syndrome coronavirus 2 (SARS-CoV-2), has resulted in over 780 million confirmed cases and 7 million deaths worldwide since its emergence in 2020 [1–3]. While the infection primarily targets the upper respiratory tract and often causes mild illness, it can progress to severe respiratory failure, requiring hospitalisation, and may result in death [3,4]. Approximately 10–20% of COVID-19 patients develop post-acute COVID-19 syndrome, also known as long COVID, characterised by persistent symptoms lasting beyond four weeks from symptoms onset without alternative diagnoses [5]. These symptoms affect multiple organ systems, including respiratory (cough, dyspnoea), cardiovascular (chest pain, palpitations), neuropsychiatric (anxiety, insomnia), musculoskeletal (joint pain, muscle weakness), dermatological (rashes, hair loss), and gastrointestinal (loss of appetite, diarrhoea) manifestations [5,6].

While several studies have investigated the impact of SARS-CoV-2 infection on the gut microbiome, findings regarding microbial diversity and taxonomic alterations have been inconsistent [3,7–10]. Gastrointestinal symptoms such as diarrhoea and nausea have been reported in 2–10% of COVID-19 patients [11,12]. The extrapulmonary dissemination of the virus is likely linked to the expression of its cellular receptor, angiotensin-converting enzyme II, in various susceptible human cells, including the small intestine and colon [8,13–15]. Therefore, the interaction between SARS-CoV-2 and angiotensin-converting enzyme II may disrupt gut microbial ecology and compromise intestinal homeostasis [3,8].

Currently, clinical management of long COVID is symptom-based, focusing primarily on rehabilitation and pharmacological interventions to alleviate symptoms [7,16]. Drugs targeting organ-specific syndromes approved for use by the Food and Drug Administration have been evaluated in early-phase proof-of-concept trials as a safe approach for long COVID within experimental clinical studies [17]. For instance, montelukast, an orally administered medication approved for asthma, has potential for alleviating long COVID-related respiratory symptoms due to its anti-inflammatory properties and efficacy in reducing oxidative stress in acute asthma [18,19]. However, commonly used non-antibiotic drugs may contribute to microbial gut dysbiosis by promoting or inhibiting the growth of specific bacterial taxa, thereby altering both microbial abundance and function [20,21]. Given that individuals with distinct gut microbiome profiles may exhibit different drug responses, we aimed to compare the gut microbiota composition of patients with long COVID and healthy controls, and to evaluate the effect of montelukast on the gut microbiota composition of patients with long COVID.

## METHODS

### Study design and sample collection

We conducted this proof-of-concept observational study at *Hospital Universitari de Bellvitge* using a cohort of 45 long COVID patients with persistent mild-moderate dyspnoea attended in primary healthcare without previous hospital admissions for SARS-CoV-2. These participants were previously enrolled in the clinical trial E-SPERANZA (ClinicalTrials.gov registration number: NCT04695704) [22], led by *Institut Universitari d’Investigació en Atenció Primària Jordi Gol*, in which they were randomised to receive treatment either with montelukast or placebo. In an independent follow-up study to investigate gut microbiome dynamics, stool samples were collected from this cohort at three time points: baseline before treatment (visit 1), after completing the 30-day treatment (visit 2), and at one year after baseline (visit 3). Patients were recruited through their primary care centres following a clinical diagnosis of long COVID, defined as a confirmed SARS-CoV-2 infection (positive RT-PCR, antigenic test, or equivalent <10 days from onset) managed in primary care, with persistent mild-to-moderate dyspnoea for more than four weeks and less than one year. All participants provided written informed consent. At enrolment, participants received a stool DNA collection & stabilisation kit (Canvax) with detailed self-collection instructions. Samples were stabilised immediately in DNA preservation buffer, transported to the microbiology laboratory and stored at -80°C until processing. A total of 106 stool samples were collected from long COVID patients (Figure S1 in the **Online Supplementary Document**): 45 samples at baseline (visit 1), 32 after treatment (visit 2), and 29 after one year (visit 3). Of the 45 participants, 23 received montelukast and 22 received placebo. We also included 45 stool samples from a previously published healthy control cohort [23], matched by age, sex and sample size (Table S1 in the **Online Supplementary Document**). Both cohorts were processed at the same institution, ensuring standardised protocols. The healthy control group, consisting of health care workers, was enrolled between November 2022 and April 2023, providing a contemporaneous geographical and temporal match to the long COVID cohort (recruited from August 2021 to March 2023). This overlap minimises potential confounding factors related to environmental changes or technical variability. The study involved two analytical approaches: a cross-sectional comparison of baseline gut microbiome composition between patients and healthy controls; and a longitudinal assessment of gut microbiome dynamics following montelukast treatment or placebo.

### DNA extraction and 16S rRNA gene amplification and sequencing

Total DNA was extracted from stool samples using the KingFisher Flex system (Thermo Fisher Scientific) and the MagMAXTM Microbiome Ultra Nucleic Acid Isolation kit, which incorporates mechanical disruption with zirconia beads, following the manufacturer’s instructions. DNA concentrations were quantified using a Qubit Flex fluorometer (Invitrogen). The V3-V4 regions of the 16S rRNA gene were amplified following the Illumina’s guidelines using the following primers: forward (5′-TCGTCGGCAGCGTCAGATGTGTATAAGAGACAGCCTACGGGNGGCWGCAG-3′) and reverse (5′-GTCTCGTGGGCTCGGAGATGTGTATAAGAGACAGGACTACHVGGGTATCTAATCC-3′). Amplicon size and integrity were verified on a TapeStation 4200 system (Agilent Technologies) using the D1000 ScreenTape assay. Libraries were quantified with Qubit (Invitrogen), pooled in equimolar amounts using a Sciclone G3 NGSx Workstation (PerkinElmer) and sequenced on an Illumina MiSeq platform (2x300).

### Bioinformatic and statistical analysis for 16S rRNA gene sequencing

Raw 16S rRNA FASTQ files were processed in QIIME2 [24] for quality control, including removal of index sequences and trimming of low-quality bases. Forward and reverse reads were merged, denoised, and clustered into amplicon sequence variants using DADA2 [25]. Taxonomic assignment was performed against the SILVA 138 reference database [26]. Amplicon sequence variants were further filtered using Phyloseq (R-Studio) [27] to remove potential contaminants (Eukaryota, Archaea, mitochondria, and chloroplasts) and aggregated at the genus level. Sequences were rarefied before alpha diversity analysis to account for uneven sequencing depth.

We verified statistical assumptions, including normality and homogeneity of variance, using the Shapiro–Wilk and Levene’s tests, respectively. For the cross-sectional analysis, we used Student’s *t*-test for parametric data and the Wilcoxon rank-sum used for non-parametric distributions. For longitudinal comparisons, we performed paired analyses to evaluate changes within the same individual over time, using the paired Student’s *t*-test or the Wilcoxon signed-rank test, as appropriate. To account for multiple comparisons in these longitudinal assessments, we adjusted *P*-values using the Bonferroni correction.

We assessed alpha diversity using the Shannon index. For beta diversity, we normalised microbial abundances to compositional data *via* total sum scaling transformation, and we evaluated community structure using Bray–Curtis, visualised *via* principal coordinate analysis and further analysed by permutational multivariate analysis of variance. We calculated relative abundances at all taxonomic levels, and analysed differentially abundant taxa using the linear discriminant effect size analysis (LEfSe) method [28]. We set the significance level at 0.05 (two-sided) for all statistical tests.

## RESULTS

### Long COVID characterisation: cross-sectional analysis

**Figure 1:**
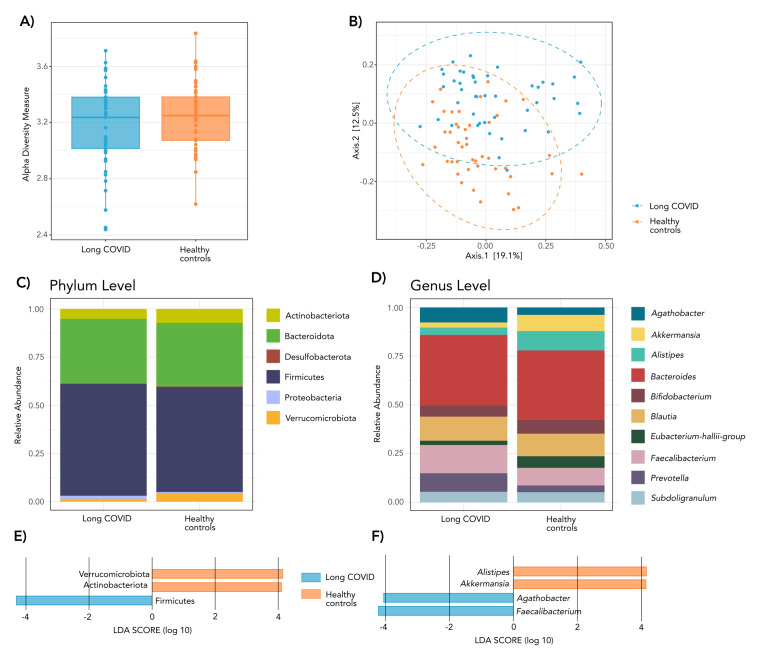
Cross-sectional analysis of the gut microbiome, relative microbial abundances and differentially enriched taxa between long COVID patients and healthy subjects. Comparison includes baseline samples from long COVID patients (n = 45) and healthy controls (n = 45). **Panel A.** Alpha diversity (Shannon index boxplots). **Panel B.** Beta diversity (principal coordinate analysis plot based on Bray–Curtis distance). Relative abundance at the phylum level (**Panel C**) and genus level (**Panel D**) in long COVID patients and healthy controls. Linear discriminant effect size analysis at the phylum level (**Panel E**) and genus level (**Panel F**) showing taxa with significant differential abundance based on the linear discriminant analysis score.

We performed a cross-sectional comparison between 45 baseline stool samples and 45 age- and sex-matched healthy control samples to investigate potential alterations in the gut microbiome of long COVID patients (Table S1 in the **Online Supplementary Document**). While alpha diversity analysis revealed no significant differences in the Shannon index, indicating comparable microbial richness and evenness (**Figure 1**, Panel A), beta diversity analysis based on Bray–Curtis dissimilarity showed a clear separation between groups (**Figure 1**, Panel B). Statistical significance was confirmed by PERMANOVA (ADONIS) testing, which revealed that the gut microbial composition of long COVID patients differed significantly from that of healthy controls (*R^2^* = 0.081, *P*-value = 0.001). This indicates that disease status explains 8.1% of the total microbial community variation. This finding was further supported by a test of multivariate homogeneity of group dispersions, which showed no significant differences between groups (*P*-value >0.05), confirming that the observed separation is driven by a true compositional shift rather than differences in group dispersion.

At the phylum level, both groups presented a typical gut microbiota dominated by Firmicutes and Bacteroidota, with smaller proportions of Actinobacteria, Proteobacteria, and Verrucomicrobiota (**Figure 1**, Panel C). Discriminant analysis revealed compositional differences between groups. The LEfSe identified Verrucomicrobiota and Actinobacteriota as significantly enriched in healthy controls, whereas Firmicutes were more abundant in long COVID patients (**Figure 1**, Panel E). Genus-level profiling provided further resolution of these patterns. The ten most prevalent genera across all samples included *Bacteroides*, followed by *Faecalibacterium* and *Blautia* as dominant taxa (**Figure 1**, Panel D). The LEfSe further identified taxa contributing to intergroup variability: *Alistipes* and *Akkermansia* were significantly enriched in healthy controls, while *Agathobacter* and *Faecalibacterium* were more abundant in long COVID patients (**Figure 1**, Panel F).

### Longitudinal long COVID analysis

Since long COVID patients were randomly assigned to receive either placebo or montelukast, we performed a longitudinal characterisation of the gut microbiome dynamics to explore whether treatment induced compositional or structural changes over time.

**Figure 2:**
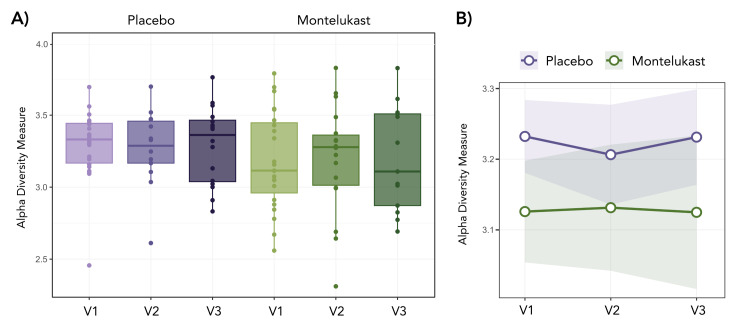
Longitudinal analysis of alpha diversity in long COVID patients treated with montelukast or placebo. **Panel A.** Alpha diversity (Shannon index) at visit 1 (baseline), visit 2 (30 days), and visit 3 (one year) in the placebo and montelukast groups. **Panel B.** Interaction plot showing the longitudinal trend of alpha diversity measures between treatment arms across the three time points.

Alpha diversity analyses showed no significant temporal variation in richness and evenness, indicating that microbial diversity remained stable throughout the study period in both placebo and montelukast groups (**Figure 2**). Similarly, beta diversity analysis showed no significant longitudinal changes within groups, suggesting preservation of the core microbial community structure regardless of treatment (Figure S2 in the **Online Supplementary Document**).

**Figure 3:**
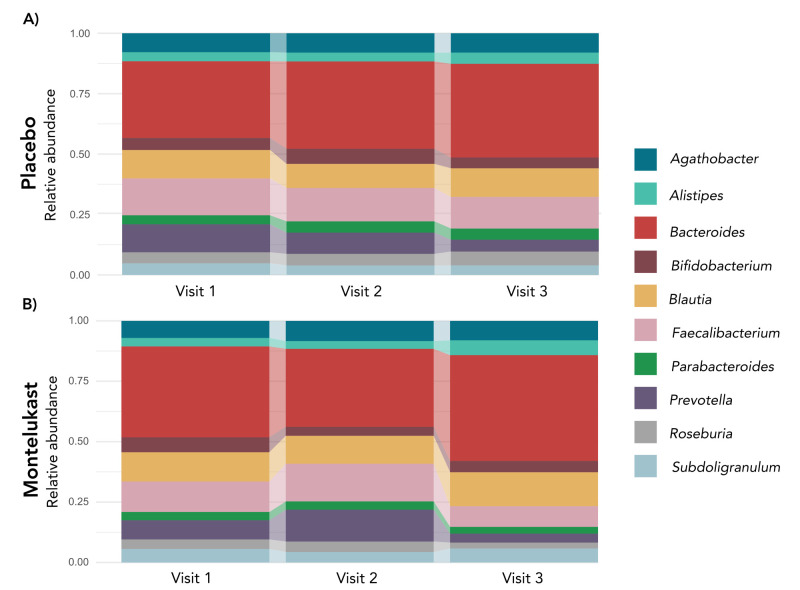
Longitudinal relative abundance of the top 10 genera in long COVID patients. Upper panels represent the placebo group and lower panels represent the montelukast group across the three study time points: visit 1 (baseline), visit 2 (30 days), and visit 3 (one year).

At the compositional level, phylum relative abundances remained largely stable across visits in both study arms. However, at the genus level, subtle temporal trends were observed among the most abundant taxa. *Bacteroides* dominated both groups, particularly the placebo group, where its relative abundance progressively increased from V1 to V3, accompanied by a concomitant decrease in *Prevotella*. In contrast, the montelukast group did not follow this trajectory, showing a transient decrease in *Bacteroides* abundance at V2 that interrupted the upward trend seen in the placebo group (**Figure 3**).

Looking into detail at these two genera, although no statistically significant differences in relative abundance of *Bacteroides* and *Prevotella* were observed between groups at any time point, visual observation of taxonomic temporal patterns highlighted subtle, yet consistent tendencies (Figure S3 in the **Online Supplementary Document**). In the placebo group, *Bacteroides* showed a continuous increase and *Prevotella* showed a progressive decrease between baseline and the follow-up visits, suggesting a stable directional shift in these dominant genera. In contrast, the montelukast group displayed less consistent dynamics, characterised by minor fluctuations over time, including a temporary increase in *Prevotella* at V2. To note, the high variability of *Prevotella* in the placebo group compared to the montelukast-treated group.

To further assess the effect of montelukast, we compared gut microbiome composition between groups at visit 2, corresponding to the end of the 30-day treatment. LEfSe identified *Dialister* as the only genus differentiating both groups, showing higher relative abundance in the montelukast-treated patients (Figure S4 in the **Online Supplementary Document**). No other taxa were significantly enriched in either group, and alpha and beta diversity comparisons at visit 2 revealed no significant differences, indicating that overall microbial diversity and community structure remained comparable between treatment groups.

## DISCUSSION

The COVID-19 pandemic posed unprecedented global health challenges, not only during the acute phase of infection, but also through the long-term sequelae experienced by many patients with long COVID [5–7]. This clinical condition prompted an urgent search for therapeutic strategies to relieve persistent symptoms and improve quality of life [7,16,17]. While some interventions have shown partial or context-dependent benefits, others have yielded reduced efficacy. In this context, growing interest has focused on the gut microbiome as a potential modulator of persistent symptoms, although current evidence remains heterogeneous and sometimes contradictory. This study contributes to the growing body of evidence on gut microbiome alterations in long COVID by first establishing a baseline comparison with a healthy population to characterise the long COVID microbial signature, and subsequently evaluating the impact of short-term montelukast treatment on these microbial profiles.

Our findings support the growing body of evidence indicating that the gut microbiome is altered in long COVID patients [3,8–10]. In our cohort, this condition was characterised by a distinct restructuring of the microbial community composition. Specifically, our analysis revealed significant shifts in beta diversity that clearly separate long COVID patients from healthy controls, with disease status explaining 8.1% of the total community variance (*R^2^* = 0.081). This clustering is driven by substantial differences in taxonomic profiles rather than internal group variability, as confirmed by the homogeneity of dispersions, supporting the identification of a specific microbial signature associated with long COVID in these patients. Furthermore, we observed that alpha diversity remained stable, indicating that overall microbial richness was not depleted. While SARS-CoV-2 interacts with gastrointestinal epithelial cells, these interactions do not invariably result in substantial loss of taxa, as is often reported in acute SARS-CoV-2 infection [29]; instead, these results imply that the observed dysbiosis in long COVID is qualitative rather than quantitative.

This realignment was also evident at the taxonomic level. At the phylum level, both groups had a typical gut microbiota, which was dominated by Firmicutes and Bacteroidota [30–32]. In our cohort, the three most prevalent genera were *Bacteroides*, *Faecalibacterium*, and *Blautia*. This reflects a core community structure that is preserved in long COVID patients [30,33]. In contrast, genera such as *Clostridium*, *Eubacterium*, and *Ruminococcus* were less abundant across all samples, possibly due to interindividual variability and external influences, such as diet, medication, or lifestyle [30–32,34,35]. Despite these commonalities, LEfSe discriminant analysis identified specific taxa contributing to the observed intergroup differences. Healthy controls were characterised by an enrichment of the phyla Verrucomicrobiota and Actinobacteriota, as well as the genera *Akkermansia* and *Alistipes*. Conversely, long COVID patients showed a higher abundance of Firmicutes, driven by an enrichment of genera such as *Agathobacter* and *Faecalibacterium*. These findings reinforce the idea that long COVID microbial restructuring is not defined by a generalised loss of key commensals but rather by a systemic reconfiguration of the microbial community.

Longitudinal analysis further confirmed overall stability of gut microbial diversity over time. The inverse relationship between *Bacteroides* and *Prevotella*, commonly associated with metabolic and inflammatory phenotypes [35,36], was also reflected in our cohort, suggesting group-specific trajectories after treatment. The temporal dynamics of the *Bacteroides/Prevotella* association diverged between groups. In the placebo group, *Bacteroides* abundance progressively increased while *Prevotella* declined, reflecting a gradual shift toward a *Bacteroides*-enriched state. In contrast, the montelukast group interrupted this trajectory by inducing a transient reduction in *Bacteroides* after the 30-day treatment. This *Bacteroides* reduction pattern resembles findings from both culture-based and metagenomic studies of antimicrobial exposure, where short-term disruptions to the gut microbiota, with a decline in *Bacteroides* alongside diversity loss are commonly observed [37]. However, in our study, montelukast caused a reduction in *Bacteroides* without a broad disruption of the gut microbial community. These changes are consistent with a drug-associated modulation of community dynamics rather than a simple time-dependent drift, aligning with evidence for taxon-specific effects of non-antibiotic compounds [20,21].

The LEfSe analysis combining cross-sectional and intervention data identified several differentially abundant taxa. When comparing long COVID patients *vs*. healthy controls, *Alistipes* and *Akkermansia* were enriched in healthy controls, while *Agathobacter* and *Faecalibacterium* were more abundant in long COVID patients. This pattern partially contrasts with acute/post-acute reports of short-chain fatty acids-producers depletion. *Akkermansia muciniphila*, a mucin-degrader promoting gut barrier function, is generally linked to favourable inflammatory and metabolic profiles [38] and *Alistipes* species exhibit context-dependent roles, with higher abundances previously associated with favourable acute COVID-19 recovery trajectories [39]. In contrast, *Faecalibacterium*, a major butyrate producer with anti-inflammatory activity [40], shows species-specific behaviours. For instance, *F. prausnitzii* enrichment has been connected to neurological long COVID symptoms such as memory loss, poor concentration, and insomnia [41]. When examining montelukast-treated vs placebo groups, *Dialister* was the only genus enriched in the treatment arm. This genus has been associated with pro-inflammatory conditions [42] potentially reflecting a transient inflammatory or immunomodulatory response rather than a sustained microbiome alteration. Taken together, the LEfSe findings suggest that taxon-specific fluctuations, rather than broad compositional shifts, occur both across disease states and following short-term drug exposure.

However, these results should be interpreted with caution, and several limitations should be acknowledged. Given the proof-of-concept nature of this study, the modest sample size limits statistical power, potentially obscuring the detection of subgroup-specific effects, particularly at lower taxonomic ranks. Clinical heterogeneity, including prior treatmentsand diet and lifestyle factors, may further contribute to variability. Although we applied specific exclusion criteria for major chronic comorbidities to ensure a more homogeneous cohort, we acknowledge that the lack of detailed metadata remains a potential source of bias. Additionally, the short intervention period captures only early microbial responses to montelukast, possibly missing delayed or cumulative effects. The lack of baseline samples prior to long COVID prevents confirming whether treatment or time promotes trends toward microbiome reversion.

## CONCLUSIONS

These results suggest that long COVID involves significant structural reorganisation of the gut ecosystem rather than depletion of its fundamental components. The condition appears to be marked by a compositional shift and quantitative re-equilibration of key microbial taxa, most notably within the phylum Firmicutes, specifically the *Faecalibacterium* and *Agathobacter* genera, indicating an altered intestinal homeostatic balance even when overall species richness is maintained. These findings suggest a qualitative transformation of the microbiota, where the core community’s identity remains present, but its internal proportions are disrupted. Furthermore, short-term montelukast administration seems to exert transient effects at the genus level without causing broader microbial landscape destabilisation. These targeted changes highlight the complexity of gastrointestinal involvement in long COVID, where the microbial architecture undergoes a persistent realignment. Extended follow-up and integrative functional analysis are essential to determine if these shifts influence host-microbiome interactions and long-term recovery.

## Additional material


Online Supplementary Document

## References

[1] World Health Organization. WHO COVID-19 dashboard – Number of COVID-19 deaths reported to WHO. Available: https://data.who.int/dashboards/covid19/deaths. Accessed: 15 December 2025.

[2] World Health Organization. COVID-19 cases – World. Available: https://data.who.int/dashboards/covid19/cases. Accessed: 15 December 2025.

[3] Zuo T Zhang F Lui GCY Yeoh YK Li AYL Zhan H Alterations in Gut Microbiota of Patients With COVID-19 During Time of Hospitalization. Gastroenterology. 2020;159:944–955.e8. 10.1053/j.gastro.2020.05.048 32442562 PMC7237927

[4] Álvarez J Fernández Real JM Guarner F Gueimonde M Rodríguez JM Saenz de Pipaon M Microbiota intestinal y salud. Gastroenterol Hepatol. 2021;44:519–35. 10.1016/j.gastrohep.2021.01.009 33652061

[5] Davis HE McCorkell L Vogel JM Topol EJ Long COVID: major findings, mechanisms and recommendations. Nat Rev Microbiol. 2023;21:133–46. 10.1038/s41579-022-00846-2 36639608 PMC9839201

[6] Yong SJ Long COVID or post-COVID-19 syndrome: putative pathophysiology, risk factors, and treatments. Infect Dis (Lond). 2021;53:737–754. 10.1080/23744235.2021.1924397 34024217 PMC8146298

[7] Zhang F Lau RI Liu Q Su Q Chan FKL Ng SC Gut microbiota in COVID-19: key microbial changes, potential mechanisms and clinical applications. Nat Rev Gastroenterol Hepatol. 2023;20:323–37. 10.1038/s41575-022-00698-4 36271144 PMC9589856

[8] Gu S Chen Y Wu Z Chen Y Gao H Lv L Alterations of the Gut Microbiota in Patients with COVID-19 or H1N1 Influenza. Clin Infect Dis. 2020;71:2669–78. 10.1093/cid/ciaa709 32497191 PMC7314193

[9] Righi E Dalla Vecchia I Auerbach N Morra M Górska A Sciammarella C Gut Microbiome Disruption Following SARS-CoV-2: A Review. Microorganisms. 2024;12:131. 10.3390/microorganisms12010131 38257958 PMC10820238

[10] Zhou J Yang X Yang Y Wei Y Lu D Xie Y Human microbiota dysbiosis after SARS-CoV-2 infection have the potential to predict disease prognosis. BMC Infect Dis. 2023;23:841. 10.1186/s12879-023-08784-x 38031010 PMC10685584

[11] Guo M Tao W Flavell RA Zhu S Potential intestinal infection and faecal–oral transmission of SARS-CoV-2. Nat Rev Gastroenterol Hepatol. 2021;18:269–83. 10.1038/s41575-021-00416-6 33589829 PMC7883337

[12] Zhang H Kang Z Gong H Xu D Wang J Li Z Digestive system is a potential route of COVID-19: an analysis of single-cell coexpression pattern of key proteins in viral entry process. Gut. 2020;69:1010–8. 10.1136/gutjnl-2020-320953

[13] Ren Z Wang H Cui G Lu H Wang L Luo H Alterations in the human oral and gut microbiomes and lipidomics in COVID-19. Gut. 2021;70:1253–1265. 10.1136/gutjnl-2020-323826 33789966 PMC8042598

[14] Villapol S Gastrointestinal symptoms associated with COVID-19: impact on the gut microbiome. Transl Res. 2020;226:57–69. 10.1016/j.trsl.2020.08.004 32827705 PMC7438210

[15] Zuo T Wu X Wen W Lan P Gut Microbiome Alterations in COVID-19. Genomics Proteomics Bioinformatics. 2021;19:679–88. 10.1016/j.gpb.2021.09.004 34560321 PMC8478109

[16] Koc HC Xiao J Liu W Li Y Chen G Long COVID and its Management. Int J Biol Sci. 2022;18:4768–80. 10.7150/ijbs.75056 35874958 PMC9305273

[17] Peluso MJ Deeks SG Mechanisms of long COVID and the path toward therapeutics. Cell. 2024;187:5500–29. 10.1016/j.cell.2024.07.054 39326415 PMC11455603

[18] Barré J Sabatier JM Annweiler C Montelukast Drug May Improve COVID-19 Prognosis: A Review of Evidence. Front Pharmacol. 2020;11:1344. 10.3389/fphar.2020.01344 33013375 PMC7500361

[19] Fidan C Aydoğdu A As a potential treatment of COVID-19: Montelukast. Med Hypotheses. 2020;142:109828. 10.1016/j.mehy.2020.109828 32416408 PMC7211747

[20] Weersma RK Zhernakova A Fu J Interaction between drugs and the gut microbiome. Gut. 2020;69:1510–9. 10.1136/gutjnl-2019-320204 32409589 PMC7398478

[21] Moreno del Castillo MC Valladares-García J Halabe-Cherem J Microbioma humano. Revista de la Facultad de Medicina (México). 2018;61:7–19. 10.22201/fm.24484865e.2018.61.6.02

[22] Mera-Cordero F Bonet-Monne S Almeda-Ortega J García-Sangenís A Cunillera-Puèrtolas O Contreras-Martos S Double-blind placebo-controlled randomized clinical trial to assess the efficacy of montelukast in mild to moderate respiratory symptoms of patients with long COVID: E-SPERANZA COVID Project study protocol. Trials. 2022;23:19. 10.1186/s13063-021-05951-w 34991703 PMC8733792

[23] Bonilla-Moreno M Medina-Gómez C Guevara-Núñez D Saiz-Escobedo L Martí S Domínguez MÁ Assessing healthcare workers as potential stool donors for faecal microbiota transplantation: a cross-sectional study of antimicrobial-resistant gut bacteria and enteropathogenic micro-organisms. J Hosp Infect. 2025;165:153–62. 10.1016/j.jhin.2025.09.004 40997947

[24] Bolyen E Rideout JR Dillon MR Bokulich NA Abnet CC Al-Ghalith GA Reproducible, interactive, scalable and extensible microbiome data science using QIIME 2. Nat Biotechnol. 2019;37:852–7. 10.1038/s41587-019-0209-9 31341288 PMC7015180

[25] Callahan BJ McMurdie PJ Rosen MJ Han AW Johnson AJA Holmes SP DADA2: High-resolution sample inference from Illumina amplicon data. Nat Methods. 2016;13:581–3. 10.1038/nmeth.3869 27214047 PMC4927377

[26] Quast C Pruesse E Yilmaz P Gerken J Schweer T Yarza P The SILVA ribosomal RNA gene database project: improved data processing and web-based tools. Nucleic Acids Res. 2013;41 D1:D590–6. 10.1093/nar/gks1219 23193283 PMC3531112

[27] McMurdie PJ Holmes S phyloseq: An R package for reproducible interactive analysis and graphics of microbiome census data. PLoS One. 2013;8:e61217. 10.1371/journal.pone.0061217 23630581 PMC3632530

[28] Segata N Izard J Waldron L Gevers D Miropolsky L Garrett WS Metagenomic biomarker discovery and explanation. Genome Biol. 2011;12:R60. 10.1186/gb-2011-12-6-r60 21702898 PMC3218848

[29] Wang M Zhang Y Li C Chang W Zhang L The relationship between gut microbiota and COVID-19 progression: new insights into immunopathogenesis and treatment. Front Immunol. 2023;14:1180336. 10.3389/fimmu.2023.1180336 37205106 PMC10185909

[30] Gomaa EZ Human gut microbiota/microbiome in health and diseases: a review. Antonie van Leeuwenhoek. 2020;113:2019–40. 10.1007/s10482-020-01474-7 33136284

[31] Hou K Wu ZX Chen XY Wang JQ Zhang D Xiao C Microbiota in health and diseases. Signal Transduct Target Ther. 2022;7:135. 10.1038/s41392-022-00974-4 35461318 PMC9034083

[32] Van Hul M Cani PD Petitfils C De Vos WM Tilg H El-Omar EM What defines a healthy gut microbiome? Gut. 2024;73:1893. 10.1136/gutjnl-2024-333378 39322314 PMC11503168

[33] Sasso JM Ammar RM Tenchov R Lemmel S Kelber O Grieswelle M Gut Microbiome–Brain Alliance: A Landscape View into Mental and Gastrointestinal Health and Disorders. ACS Chem Neurosci. 2023;14:1717–63. 10.1021/acschemneuro.3c00127 37156006 PMC10197139

[34] Borrego-Ruiz A Borrego JJ Human gut microbiome, diet, and mental disorders. Int Microbiol. 2025;28:1–15. 10.1007/s10123-024-00518-6 38561477 PMC11775079

[35] Dong TS Guan M Mayer EA Stains J Liu C Vora P Obesity is associated with a distinct brain-gut microbiome signature that connects Prevotella and Bacteroides to the brain’s reward center. Gut Microbes. 2022;14:2051999. 10.1080/19490976.2022.2051999 35311453 PMC8942409

[36] Stanislawski MA Dabelea D Lange LA Wagner BD Lozupone CA Gut microbiota phenotypes of obesity. NPJ Biofilms Microbiomes. 2019;5:18. 10.1038/s41522-019-0091-8 31285833 PMC6603011

[37] Jakobsson HE Jernberg C Andersson AF Sjölund-Karlsson M Jansson JK Engstrand L Short-term antibiotic treatment has differing long-term impacts on the human throat and gut microbiome. PLoS One. 2010;5:e9836. 10.1371/journal.pone.0009836 20352091 PMC2844414

[38] Ye WY Cai Y *Akkermansia muciniphila*: a microbial guardian against oxidative stress-gut microbiota crosstalk and clinical prospects. J Transl Med. 2025;23:1169. 10.1186/s12967-025-07149-z 41137026 PMC12551360

[39] Hazan S Stollman N Bozkurt HS Dave S Papoutsis AJ Daniels J Lost microbes of COVID-19: Bifidobacterium, Faecalibacterium depletion and decreased microbiome diversity associated with SARS-CoV-2 infection severity. BMJ Open Gastroenterol. 2022;9:e000871. 10.1136/bmjgast-2022-000871 35483736 PMC9051551

[40] Sokol H Pigneur B Watterlot L Lakhdari O Bermúdez-Humarán LG Gratadoux JJ Faecalibacterium prausnitzii is an anti-inflammatory commensal bacterium identified by gut microbiota analysis of Crohn disease patients. Proc Natl Acad Sci U S A. 2008;105:16731–6. 10.1073/pnas.0804812105 18936492 PMC2575488

[41] Xie Q Ni J Guo W Ding C Wang F Wu Y Two-year follow-up of gut microbiota alterations in patients after COVID-19: from the perspective of gut enterotype. Microbiol Spectr. 2025;13:e0277424. 10.1128/spectrum.02774-24 40207964 PMC12054050

[42] Valles-Colomer M Falony G Darzi Y Tigchelaar EF Wang J Tito RY The neuroactive potential of the human gut microbiota in quality of life and depression. Nat Microbiol. 2019;4:623–32. 10.1038/s41564-018-0337-x 30718848

