# Supplementary material for: Gut microbiome shift in long COVID: impact of disease and montelukast treatment
Source: J Glob Health. 2026 May 15;16:04164. doi: 10.7189/jogh.16.04164 (PMC13178059; doi:10.7189/jogh.16.04164)
Supplement: Online Supplementary Document [file jogh-16-04164-s001.pdf]

Supplement to: Camps-Massa P, Pérez-Mormeneu J, Guevara-Núñez D, Saiz-Escobedo L, Calatayud L, González-Díaz A, Sanllorente A, Vicens-Zygmunt V, Santos S, Morros R, Slavador-González B, Domínguez MA, Martí S. Gut microbiome shift in long COVID: impact of disease and montelukast treatment. J Glob Health. 2026;16:04164.

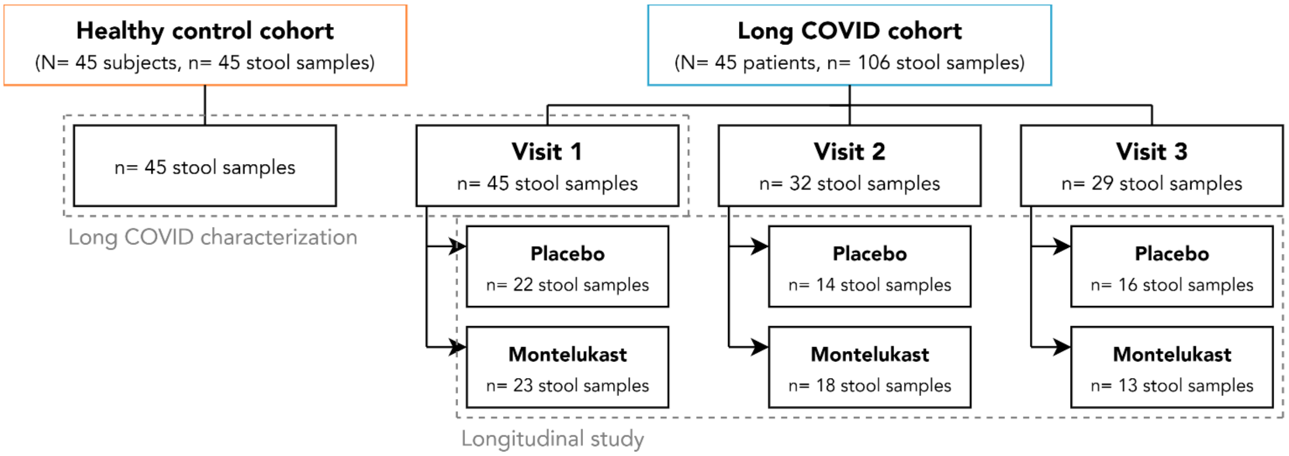

Figure S1. Flowchart of the clinical study. Stool samples from Long COVID patients were obtained at three time points: baseline (V1), 30 days post-treatment (V2), and one-year follow-up (V3). A cohort of healthy subjects was included as a control group for cross-sectional microbiome characterization.

Table S1. Age and sex distribution of the healthy control cohort and the Long-COVID patient cohort stratified by placebo or Montelukast treatment.

	Long-COVID (n = 45)	Placebo (n = 22)	Montelukast (n = 23)	Healthy controls (n = 45)
**Female patients**, n (%)	8 (17,8%)	5 (22,7%)	3 (13,0%)	8 (17,8%)
**Age**, median [IQR]	47 [41- 52]	49 [41,3-53,5]	46 [40,5-49,5]	44 [37-56]

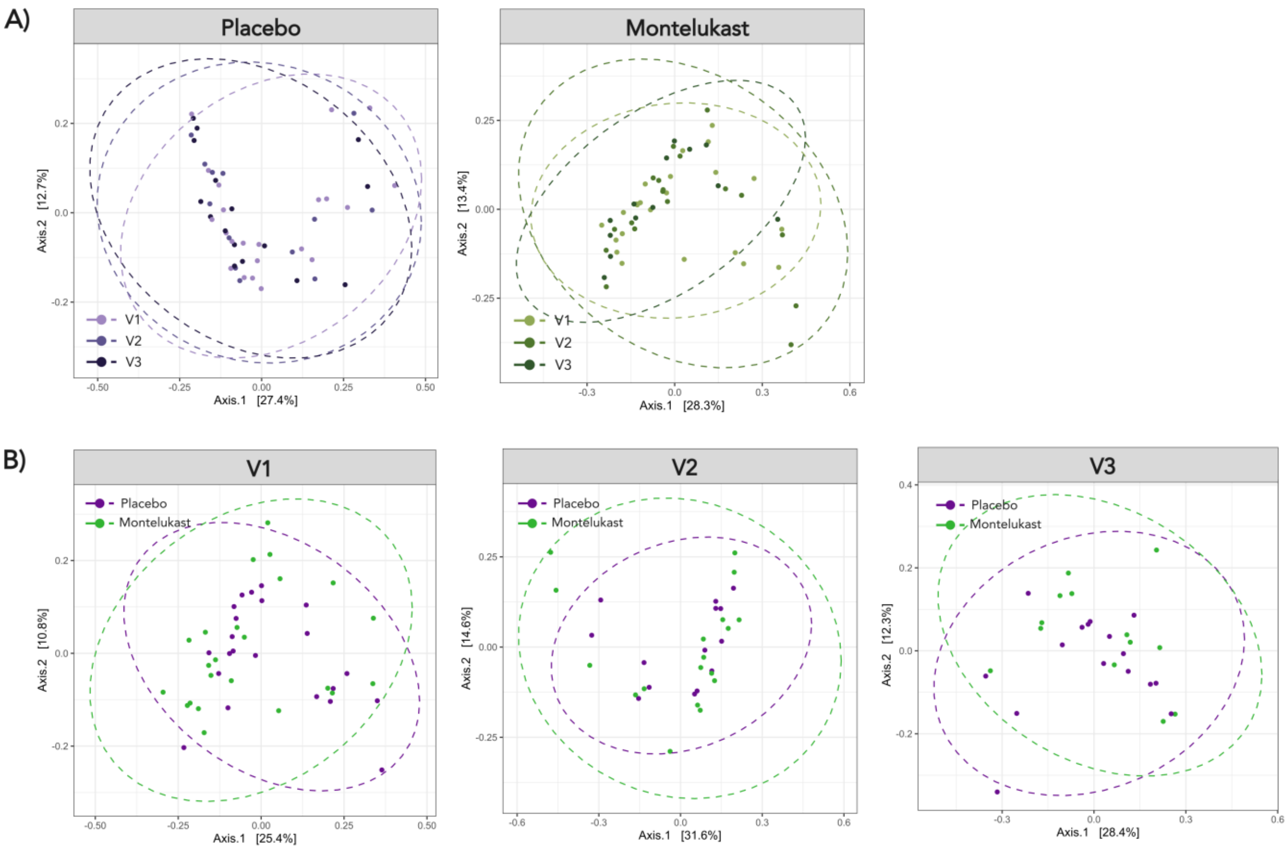

Figure S2. Longitudinal analysis of gut microbial diversity in Long COVID patients. A) Beta diversity (Bray-Curtis distance) across time points within the placebo and Montelukast groups. **B)** Beta diversity (Bray-Curtis distance) comparing the placebo and Montelukast groups at each individual time point.

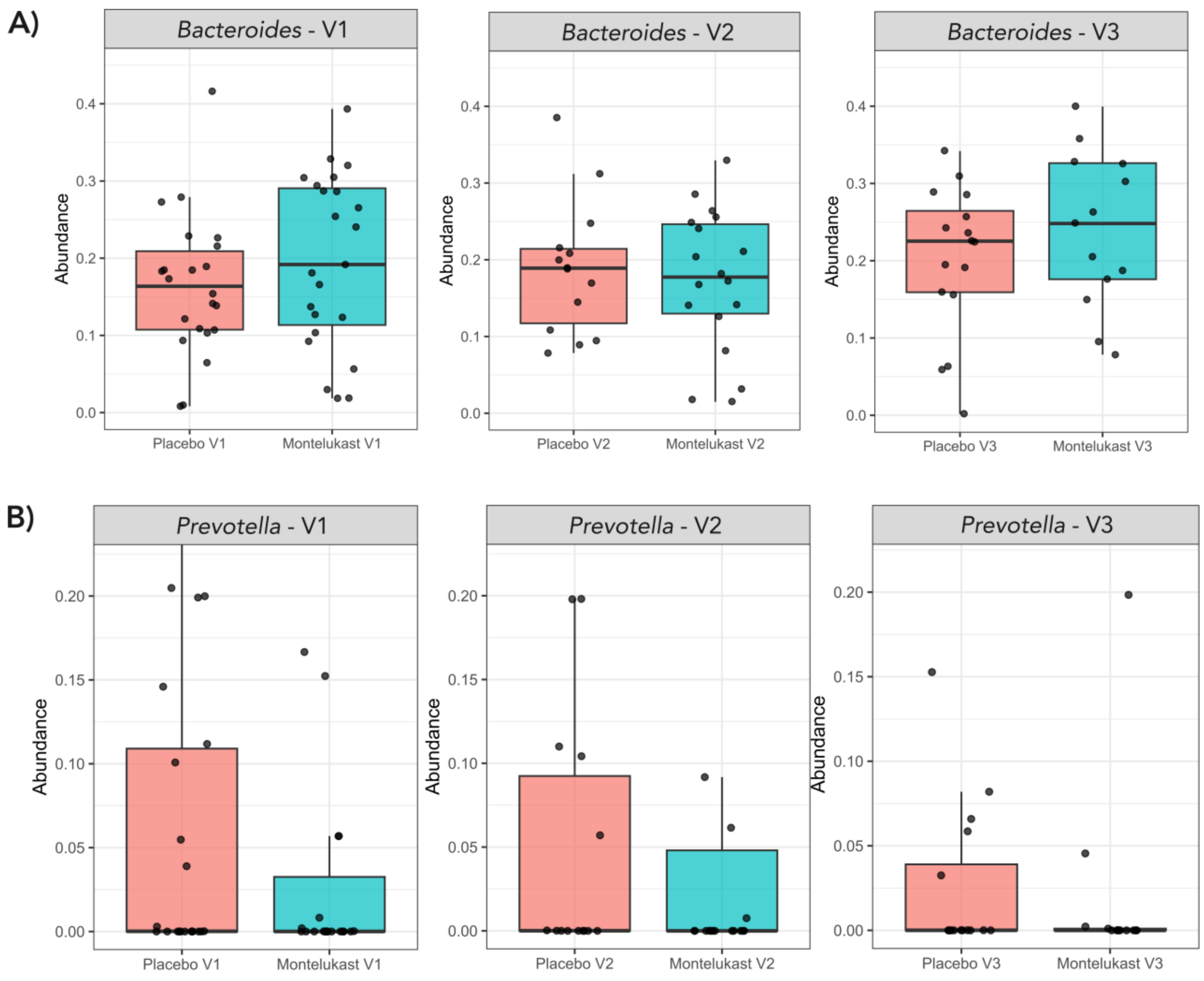

Figure S3. **Longitudinal fluctuation in relative abundance of *Bacteroides* and *Prevotella* in the gut microbiome. A)** Relative abundance of *Bacteroides* and **B)** relative abundance of *Prevotella* across the three study visits: V1 (before treatment), V2 (after completing the 30-day treatment), and V3 (one year after baseline) for both treatment groups.

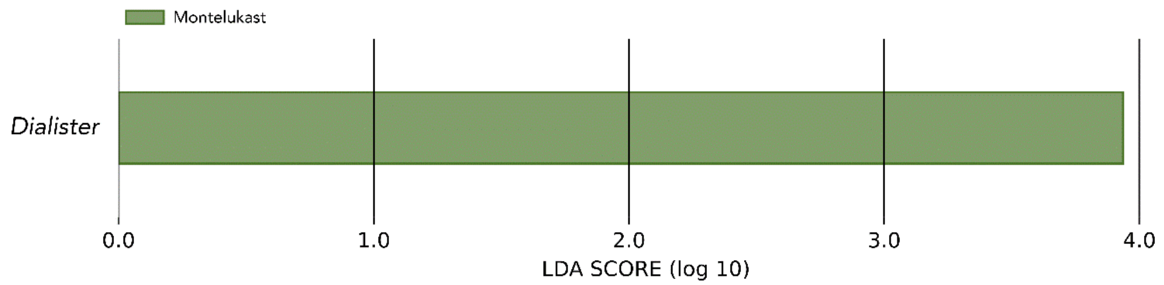

Figure S4. Differentially enriched taxa in Long COVID patients following Montelukast treatment. LEfSe analysis at the genus level comparing the placebo and Montelukast groups at Visit 2 (30 days post-treatment). Taxa are identified based on the Linear Discriminant Analysis (LDA) score.

E-SPERANZA study group members: Jesús Almeda-Ortega, Atenció Primària Institut Català de la Salut – IDIAPJGol; Sara Contreras-Martos, Atenció Primària Institut Català de la Salut – IDIAPJGol; Sara Bonet Monne, Atenció Primària Institut Català de la Salut – IDIAPJGol; Gemma Alvarez Muñoz, Atenció Primària Institut Català de la Salut – IDIAPJGol; Francisco Mera-Cordero, EAP El Pla, Sant Feliu de Llobregat; Nancy Eydis Castillo Elinan, EAP El Pla, Sant Feliu de Llobregat; Beatriz Navarro Martín, EAP El Pla, Sant Feliu de Llobregat; Maria Jesús Gallardo Guerra, EAP Jaume Soler, Cornellà de Llobregat; Jenifer Botanes Iglesias, EAP Jaume Soler, Cornellà de Llobregat; Sofía Centeno Manotas, EAP Jaume Soler, Cornellà de Llobregat; Maria Antonia Coll Bosch, EAP Doctor Martí i Julià, Cornellà de Llobregat; Cristina Armengol Mercade, EAP Vinyets, Sant Boi de Llobregat; Elena Barquero Bardon, EAP Vinyets, Sant Boi de Llobregat; Marta Via Vidal, EAP Molí Nou, Sant Boi de Llobregat; Encarnación Magraner Esteve, EAP Molí Nou, Sant Boi de Llobregat; Maria Teresa Ortiz Lupiañez, EAP Molí Nou, Sant Boi de Llobregat; Arnau Segura Anducas, EAP Corbera, Corbera de Llobregat; Francisco Javier Calero Ribera, EAP 17 de Setembre, El Prat de Llobregat; Cristina López Ramírez, EAP 17 de Setembre, El Prat de Llobregat; Alex Trepat González, CAP Just Oliveras, L'Hospitalet de Llobregat; Josefa Pérez Ruiz, CAP Just Oliveras, L'Hospitalet de Llobregat; Isabel Zamora Casas, EAP Santa Eulàlia Sud, L'Hospitalet de Llobregat; Carles Rubio Ripollès, EAP Florida, L'Hospitalet de Llobregat; Ramon Monfà Escolà, Atenció Primària Institut Català de la Salut – IDIAPJGol; Ana García Sangenís, Atenció Primària Institut Català de la Salut – IDIAPJGol; Oriol Cunillera Puértolas, Atenció Primària Institut Català de la Salut – IDIAPJGol; Cristina Miranda Jiménez, Atenció Primària Institut Català de la Salut – IDIAPJGol; Anna Cortes Bosch de Basea, Hospital Universitari de Bellvitge, ICS – IDIBELL; Anna Ferrer Artola, Hospital Universitari de Bellvitge, ICS – IDIBELL.
